# Adaptive Lipid Packing and Bioactivity in Membrane Domains

**DOI:** 10.1371/journal.pone.0123930

**Published:** 2015-04-23

**Authors:** Erdinc Sezgin, Theresia Gutmann, Tomasz Buhl, Ron Dirkx, Michal Grzybek, Ünal Coskun, Michele Solimena, Kai Simons, Ilya Levental, Petra Schwille

**Affiliations:** 1 Weatherall Institute of Molecular Medicine, University of Oxford, Oxford, United Kingdom; 2 Max Planck Institute for Molecular Cell Biology and Genetics, Dresden, Germany; 3 Paul Langerhans Institute Dresden of the Helmholtz Centre Munich at the University Clinic Carl Gustav Carus, TU Dresden, Dresden, Germany; 4 German Center for Diabetes Research (DZD), Neuherberg, Germany; 5 Department of Integrative Biology and Pharmacology, University of Texas Health Science Center, Houston, Texas, United States of America; 6 Max Planck Institute of Biochemistry, Martinsried-Munich, Germany; Institut Curie, FRANCE

## Abstract

Lateral compositional and physicochemical heterogeneity is a ubiquitous feature of cellular membranes on various length scales, from molecular assemblies to micrometric domains. Segregated lipid domains of increased local order, referred to as rafts, are believed to be prominent features in eukaryotic plasma membranes; however, their exact nature (i.e. size, lifetime, composition, homogeneity) in live cells remains difficult to define. Here we present evidence that both synthetic and natural plasma membranes assume a wide range of lipid packing states with varying levels of molecular order. These states may be adapted and specifically tuned by cells during active cellular processes, as we show for stimulated insulin secretion. Most importantly, these states regulate both the partitioning of molecules between coexisting domains and the bioactivity of their constituent molecules, which we demonstrate for the ligand binding activity of the glycosphingolipid receptor GM1. These results confirm the complexity and flexibility of lipid-mediated membrane organization and reveal mechanisms by which this flexibility could be functionalized by cells.

## Introduction

The membrane raft hypothesis proposes a laterally heterogeneous structure for biological membranes [1], with lipid-induced membrane domains being responsible for functional compartmentalization of cellular trafficking and signaling activities in a wide variety of cellular contexts [2,3]. However, the physicochemical nature of raft-mediated membrane heterogeneity and its direct impact on cell function remains difficult to define. One reason is that the combination of sub-resolution size and potentially transient nature of functional domains escapes most of direct detection technologies. The original and still predominant method for measuring raft composition in live cells is indirectly via their detergent resistance [4], wherein the differential solubility of membrane components to non-ionic detergents is inferred to be related to the localization of those components in raft domains. This interpretation is complicated by the unpredictable interactions between biological membranes and detergents, which can themselves induce detergent resistant residues of very different compositions [5,6], depending on the choice of detergent and solubilization conditions (temperature, time, etc.).

The other major piece of evidence in support of the raft hypothesis is the liquid-liquid phase coexistence characteristic to a wide variety of sterol-containing biomimetic membranes [7]. In these, saturated lipids and sterols condense into a liquid ordered (Lo) phase (analogous to raft domains in cells) that separates from an unsaturated lipid-rich liquid disordered (Ld) phase (non-raft analog) [8]. These biomimetic systems can be investigated in great detail under tight experimental control, but are inherently limited in complexity (most notably in protein content and lipid diversity), and therefore in their physiological relevance. The compositional limitation has only recently been overcome by the observation of liquid-liquid phase separation in giant plasma membrane vesicles (GPMVs) isolated from live cells [9].

Finally, a variety of microscopic and spectroscopic techniques have been applied to elucidate the underlying physical principles of membrane heterogeneity (reviewed in [10]), probing differential diffusion of raft molecules / domains or specific interactions between various raftophilic molecules to infer the underlying membrane structure.

Recently, it was shown that the physicochemical properties of coexisting liquid phases can be dramatically different between cell-derived membranes (GPMVs) and synthetic giant unilamellar vesicles (GUVs) [11], and that this difference can alter the respective bioactivity of membrane molecules [12]. This effect was also observable between different biologically derived membranes, in which both ordered and disordered domains can vary widely in composition, lipid packing, and stability [13]. These findings are supported by recent analysis of the time-correlated fluctuations of Laurdan generalized polarization, which suggest domains of different sizes and packing states in live cell membranes [14].

Here, we explicitly observe biomembranes sampling different lipid packing states that form coexisting domains (i.e., relatively ordered and disordered domains) with a variety of distinct compositions and functional characteristics. We first investigate the connection between membrane composition and lipid packing in synthetic liposomes and show that both the relatively ordered and disordered phase, and the difference between them, are tuned by lipid composition. We then give an example of live cells regulating the lipid packing phenotypes of their plasma membranes, accessing a variety of both ordered and disordered domains as a function of cellular activity. Finally, we find that in both synthetic and natural membranes, the tunable interdomain lipid packing disparity (i.e. the difference between coexisting domains) regulates component partitioning between domains and the functionality of membrane embedded lipidic receptors.

## Materials and Methods

### Cell culture

RBL-2H3 cells (rat, ATCC #CRL-2256) were maintained in 60% RPMI, 30% MEM and 10% FCS. Ins-1 cells [15] (rat) were cultured in RPMI 1640 supplemented with 11 mM glucose, 2 mM glutamine, 10% Serum, 10 mM HEPES (pH 7.3) and 2% Ins1 supplement (1% glutamine, 1 mM NaPyr, 50 μM beta-mercaptoethanol in PBS). Cells were seeded at least 48 hours before the experiments.

### Liposome preparation

All lipids were purchased from Avanti as chloroform solutions. Lipids were mixed at the appropriate compositions, then the chloroform was slowly evaporated under a N_2_ stream followed by complete drying under vacuum for 2 h. The resulting lipid films were hydrated in liposome buffer (150 mM NaCl, 25 mM HEPES, pH 7.4) for 30 mins, with occasional vigorous mixing by vortex. These membrane suspensions were sonicated in a bath sonicator for 2 minutes to produce small unilamellar liposomes.

### GUV preparation

GUVs were prepared as described previously [12]. Briefly, 1–5 μL of 1 mg/ml lipid solutions (in chloroform) were spread onto a platinum wire, which was then submerged into 300 mM sucrose solution in a custom-built Teflon chamber. GUV formation was induced by applying 2V at 10 Hz for 2 h. For imaging, the GUV suspensions were diluted into 300 mM glucose, which is isotonic but less dense than the sucrose solution, resulting in the GUVs settling to the bottom of the imaging chambers.

### GPMV preparation

GPMVs were prepared as described previously [9]. Cells grown to 70–80% confluency were chemically induced to form plasma membrane blebs by 25 mM paraformaldehyde and 2 mM dithiothreitol (DTT) in buffer (150 mM NaCl, 10 mM Hepes and 2 mM CaCl2 (pH 7.4)) for 2 h at 37°C. GPMVs were decanted or pipetted directly from the cellular supernatant and stored at 4°C for up to 1 day. Fluorescent lipids or dyes were added to GPMVs at a concentration of 0.1–1 μM. For imaging phase separation, vesicles were cooled to 10°C with a microscope-mounted Peltier element.

### C-Laurdan imaging and GP measurement

Vesicles were labeled with 0.4 μM and 4 μM C-laurdan for spectroscopy and two-photon microscopy, respectively. For spectroscopy, liposome suspensions were excited at 385 nm and the emission was collected between 400–550 nm. For microscopy, the Biorad 2-photon setup was used. An 800 nm Ti-Sapphire laser was used for excitation and the emission was collected using 425/50 and 525/70 filters in the ordered and disordered channels, respectively. A λ/4 plate was used to eliminate the photoselection property of C-Laurdan. The GP values were calculated as described previously [9].

### Confocal imaging and partitioning measurements

All GUV imaging was performed at 23°C while GPMVs were imaged at 10°C. Vesicles were imaged with a Zeiss LSM 510 confocal microscope in BSA-coated Labtek chambers (#1.5). 488 nm, 543 nm and 633 nm lasers were used for excitation of green, orange and red fluorophores, respectively. BP 530–550, BP 585–615 and LP 650 filters in multi-track mode were used to eliminate the cross talk. ImageJ-Line profile was used to calculate the Lo % as described in ref [9,12,16]. A line was selected which crosses the opposite sides of the equatorial plane of the GUVs or GPMVs having different phases on opposite sides. Opposite sides are chosen to eliminate the laser excitation polarization artefacts. %Lo was calculated as;

$$\%Lo\;=\;F_{Lo}/(F_{Lo}+F_{Ld})$$ (Eq 1)

where F is the fluorescence emission intensity after background (i.e. outside the vesicle) intensity subtraction. If *%Lo* > 50%, a lipid analog prefers the liquid ordered (raft) phase.

### Glucose stimulation

Ins1 cells were seeded two days before the measurements. Cells were incubated in resting (15 mM HEPES pH 7.4, 5 mM KCl, 120 mM NaCl, 24 mM NaHCO_3_, 1 mM MgCl_2_, 2 mM CaCl_2_, 1mg/ml albumin, 2.8 mM glucose) or stimulation buffer (15 mM HEPES pH 7.4, 55 mM KCl, 70 mM NaCl, 24 mM NaHCO_3_, 1 mM MgCl_2_, 2 mM CaCl_2_, 1mg/ml albumin, 25 mM glucose) before GPMV preparation. Insulin secretion was measured with radioimmunoassay. After GPMVs were prepared and collected, the amount of total membrane was further equalized by using lipid scattering, as previously described [9].

### Cholesterol / phospholipid (Chol/PL) measurements

GPMVs preparations were extracted by the Folch method using chloroform and methanol to extract membrane lipids. Chloroform solution were dried and rehydrated as described above, then split into two equal aliquots. One aliquot was used to quantify free cholesterol, using the Amplex Red kit (Life Technologies) according to manufacturer instructions. The second aliquot was used to measure phospholipid concentration by the inorganic phosphate assay.

## Results and Discussion

### Compositional tuning of lipid packing in domains of model membranes

**Fig 1:**
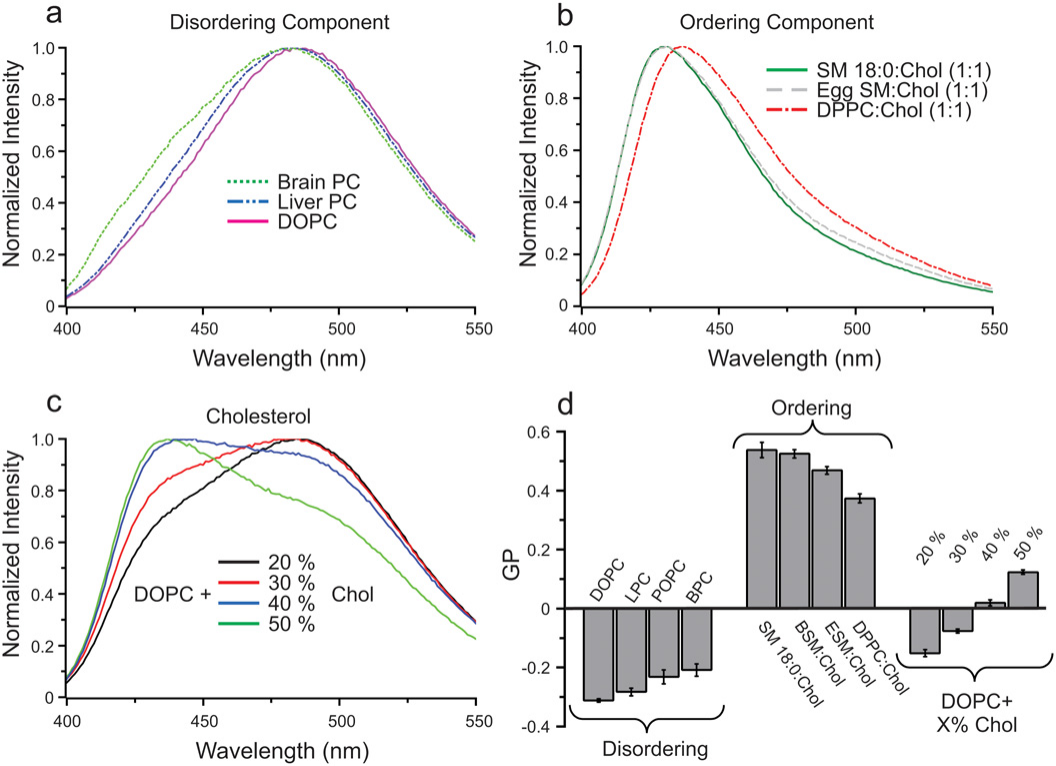
Various lipid mixtures yield a range of lipid packing states. (a) Natural PC mixtures (brain or liver PC extracts) yield liposomes with higher GP than synthetic DOPC bearing two unsaturated acyl chain. (b) Mixtures of cholesterol with natural SM (egg SM extract) and DPPC yield liposomes with lower order than cholesterol with pure SM 18:0. (c) Lipid packing of DOPC liposomes increases monotonically with increasing cholesterol. (d) Quantification of the effect of compositional variation on membrane order in one-phase liposomes.

It was recently observed that biological membranes can assume a number of distinct lipid packing states (13) and that these may correspond to domains in live cell membranes [14]. To investigate the design principles underlying differential lipid packing, we investigated the compositional determinants of membrane order in biomimetic membranes. The packing of synthetic membranes (~100 nm diameter spherical vesicles-large unilamellar vesicles (LUVs)) with various lipid mixtures was assayed by a quantitative ratiometric fluorescence assay, namely C-Laurdan spectroscopy. C-Laurdan [17] displays a membrane packing-dependent blue shift in its emission spectrum in ordered membranes, quantified as the intensity-normalized dimensionless parameter Generalized Polarization (GP: from -1 to +1, representing maximally disordered and maximally ordered membranes, respectively) [18]. Membranes designed to represent possible compositions of disordered membrane domains—i.e. comprised entirely or largely of unsaturated lipids (dioleoyl phosphatidyl choline; DOPC or natural PC mixtures, respectively)—revealed distinct physical properties based on the specific choice of “disordering component”. Natural lipid mixtures, such as liver or brain PC (LPC/BPC), formed more ordered membranes than synthetic DOPC, which is the most commonly used non-raft-mimetic lipid, and in our experiments formed an extremely disordered membrane (Fig 1a and 1d).

The observations were inverted for membranes representing Lo domains: natural saturated lipid mixtures (e.g. egg sphingomyelin; ESM) mixed with cholesterol (1:1) formed less ordered membranes than synthetic SM (18:0; SSM) (Fig 1b and 1d). Again, the most commonly used “ordering component” in model membrane studies (SSM) formed an unnaturally packed ordered domain (Fig 1b and 1d). Replacing SSM by DPPC, which has saturated acyl chains and a choline headgroup similar to SSM, led to the least ordered membranes, consistent with the role of sphingosine-mediated hydrogen bonding [19] in membrane organization. Finally, as expected, cholesterol had a progressive ordering effect on unsaturated lipid membranes, gradually increasing GP from -0.3 without cholesterol to +0.1 in a 1:1 mixture with DOPC (Fig 1c and 1d). It is important to note that C-Laurdan is an indirect probe for molecular lipid packing, whose spectral properties are determined mostly by the level of hydration in the hydrophobic/hydrophilic interfacial region of the membrane [20]. The effect on the hydrophobic core of the membrane is not explored here.

The results from these experiments are generally in line with the significant literature [7,21] on the packing of biomimetic lipid membranes, confirming that saturated lipids and cholesterol induce tightly packed/ordered membranes, while unsaturated lipids promote membrane disorder. In addition to demonstrating the possible compositional determinants of packing in biological membranes, the key conclusion from these observations is that lipid packing can be tuned to obtain a large variety of distinct states with specific physical properties even in simple, monophasic, model membranes.

**Fig 2:**
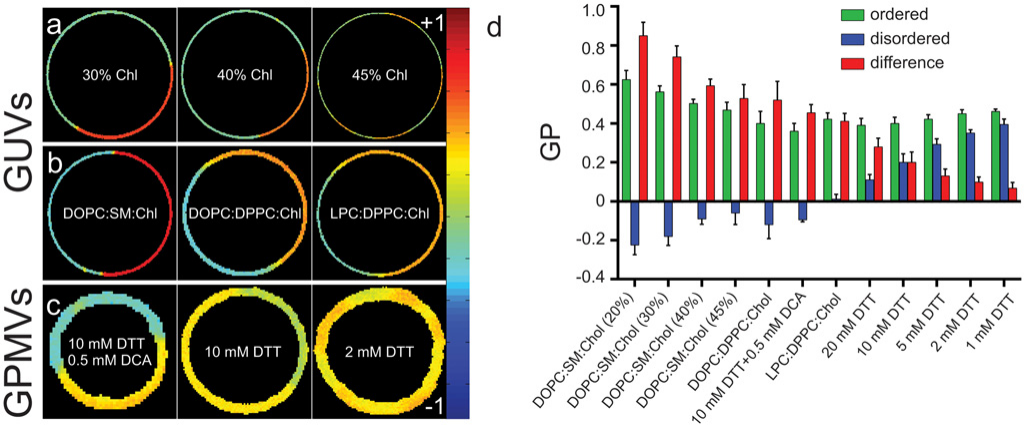
Tuning lipid packing in coexisting domains of GUVs and GPMVs. GP imaging of ordered and disordered phases in (a) DOPC:SSM:Chol GUVs with equimolar DOPC and SSM and varying Chol; (b) 2:2:1 GUVs with various ordering and disordering components; and (c) various GPMV preparations. In GPMVs, 0.5mM DCA lowers the GP of the disordered phase; [DTT] increases GP of the ordered and lowers GP of disordered. (d) Lipid packing (GP) of individual membrane domains and the differences (ΔGP) between ordered and disordered phases in phase separated membranes.

To evaluate the impact of compositional variations in systems with the bi-phasic organization of biological/biomimetic membranes [8,21,22], we prepared phase separated GUVs with various saturated/unsaturated components and cholesterol levels and imaged GP in the coexisting domains by two-photon microscopy [11,21] (Fig 2). In these systems, the compositional effects on membrane order were less predictable than in homogeneous liposomes. For example, increasing cholesterol from 30–45% decreased the GP (i.e. order) of the ordered phase while slightly increasing the GP of the disordered phase (Fig 2a), underlining the complexity of cholesterol interactions in mixed lipid systems. Varying the “ordering” and “disordering” components in phase separated GUVs also changed the order of the coexisting phases. The commonly used “raft mixture” [23] combining DOPC, sphingomyelin, and cholesterol (DOPC:SSM:Chol, 2:2:1) yielded a very tightly packed Lo phase and very loosely packed Ld phase (Fig 2b). Replacing SSM by DPPC dramatically decreased the GP of the ordered phase, in concordance with liposome experiments (Fig 1b). Similarly, replacing DOPC with natural (liver) PC slightly increased the GP of the disordered domain (Fig 2b), as predicted from LUV data. Thus, compositional variations lead to a number of distinct lipid packing states that can coexist as domains in ternary GUV mixtures.

Although these observations make inroads into defining the compositional determinants of the packing of biological membranes, such model membrane experiments are inherently limited by the compositional simplicity of model membranes. Live cell membranes are incomparably more complex than synthetic systems, due to their lipid diversity [24–26], protein content and variety, and integration with other cellular structures/systems. To determine whether our observations of variable coexisting domains are relevant for biological membranes, we imaged order in GPMVs—isolated plasma membrane vesicles that maintain the compositional complexity of their native source while still separating into distinct domains of varying order and composition [27,28]. Due to their natural origin and complexity, it is very difficult, if not impossible, to precisely control the composition of GPMVs. However, recent publications have demonstrated that their properties can be tuned by either isolation conditions (specifically [DTT]) [13] or exogenous amphiphiles, including the bile deoxycholic acid (DCA) [29] and liquid anaesthetics [30]. Consistently, we observed significant variation in lipid packing of the coexisting domains of GPMVs (Fig 2c and 2d). DCA induced a dramatic reduction in the GP of the more disordered phase, with almost no effect on the ordered domain (compare first and second row images in Fig 2c). In contrast, [DTT] increased the GP of the ordered domain while also disordering the disordered domain. Both DCA and DTT had the effect of enhancing the *difference* between the ordered and disordered domains (ΔGP). While these specific perturbations are likely not physiologically relevant, they nevertheless reveal that cell-derived plasma membranes have access to a variety of lipid packing states.

Thus, the organization / packing of coexisting domains in both synthetic and natural membranes consists of a spectrum of possible ordered and disordered states. Notably, domains in natural (GPMV) membranes are strikingly different from those of synthetic GUVs (Fig 2b–2d). The ordered phase in GPMVs is less ordered than the L_o_ domains in synthetic membranes, while the disordered phase of the cell-derived membranes is *much* more packed than the L_d_ phase of any GUV mixture measured here. This dramatic difference in ΔGP is evident in Fig 2d: whereas differences as great as 0.8 GP units were observed for synthetic membranes, unperturbed natural membranes had ΔGP < 0.1, and as low as 0.07 (Fig 2d). This trend of more native conditions promoting smaller ΔGP was maintained throughout our observations: (1) GUVs containing natural lipid mixtures (i.e. LPC) had smaller ΔGP than pure lipids (DOPC:SM:Chol); (2) less DTT yielded smaller ΔGP; (3) DCA increased ΔGP. Thus, it can be concluded that biological membranes likely maintain domains with rather small differences, consistent with recent observations of critical fluctuations in isolated PMs [30,31]. We speculate that this configuration allows cells to regulate such domains with relatively little energy input, for example by local lipid metabolism, regulated exocytosis, or cytoskeletal rearrangements. Most importantly, the data in Fig 2 emphasize the range of observable lipid packing and interdomain differences (from nearly null to 0.8 GP units) in biological and biomimetic membranes. It is possible that even smaller interdomain differences may allow phase separation; however, these were not observed with the limited resolution of GP imaging.

It is important to highlight that despite the broad range of domains observed and the presence of thousands of individual components (in GPMVs), only two microscopically distinguishable liquid phases—a relatively ordered and relatively disordered one—were ever observed in a single vesicle. We speculate that this is due to the thermodynamically equilibrated state of isolated vesicles, and that in live cells, multiple domains of different compositions could coexist within a single membrane due to active, local processes like membrane traffic, lipid turnover, enzymatic activity, etc.

An additional consideration is the likely effect of temperature on membrane packing and composition of the coexisting domains. All GUV imaging was performed at 23°C while GPMVs were imaged at 10°C. Ideally, comparisons would be made at the same reduced temperature (i.e. T—T_phase separation_) rather than the same absolute temperature, but this is practically difficult for the range of compositions employed here. The effect of temperature on packing and partitioning between domains remains an open question.

### Cells tune the physical properties of plasma membrane domains

To demonstrate the functional modulation of lipid packing in live cells, we employed a model excitable cell line (Ins1 pancreatic beta cells, which secrete insulin upon glucose stimulation) to rapidly induce cellular rearrangements and monitor the resulting changes in plasma membrane (PM) physical properties. Although the regulation of insulin secretion is intensively investigated, the physical and functional changes to the PM induced by insulin granule exocytosis have not been widely examined. PMs were isolated as GPMVs [9] and PM order was quantified during glucose stimulation (see experimental procedures for details) by both microscopic and spectroscopic evaluation of C-Laurdan emission spectra.

**Fig 3:**
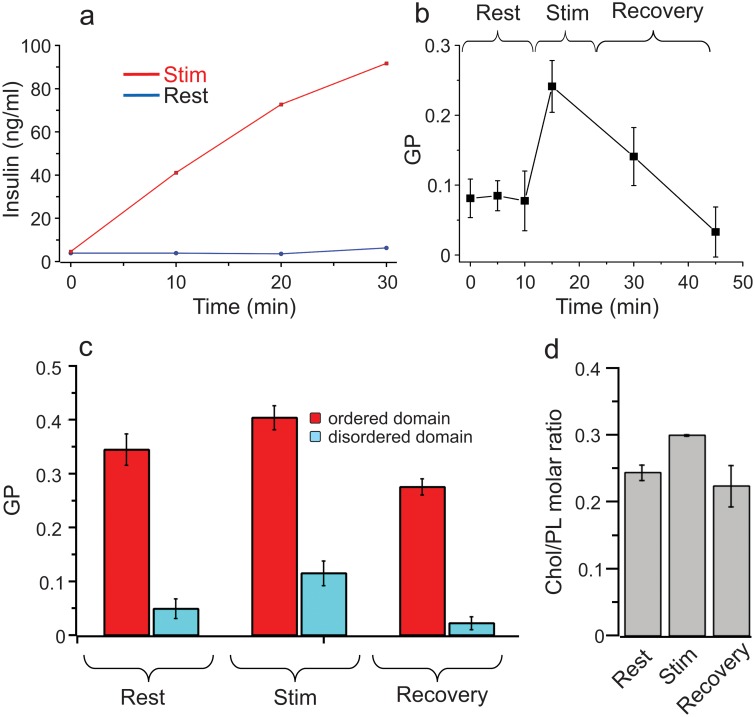
Membrane order is modulated in a domain-specific way by cellular activity. (a) Confirmation of insulin secretion induced by glucose stimulation. (b) Time course of overall PM order during glucose stimulation of beta cells. (c) C-Laurdan microscopy of GPMVs shows domain-specific changes to membrane order during the insulin stimulation time-course (p values determined with unpaired t-test). (d) Changes to PM order are driven by changes in membrane composition, with PM cholesterol (normalized to membrane PLs) increasing with glucose stimulation, and decreasing during recovery.

Shortly after glucose stimulation (confirmed by insulin secretion (Fig 3a)), the order of the beta cell PM increases sharply (Fig 3b), presumably due to exocytic fusion of insulin granule membranes with the PM. With longer stimulation, PM packing recovered with a slight overshoot of the original GP value (Fig 3b). Having observed dramatic changes to overall PM order, we investigated stimulation-induced changes to individual domains by C-Laurdan microscopy. Both raft and non-raft phases in GPMVs became more ordered during glucose stimulation, though not to the same magnitude (Fig 3c). Moreover, we observed a recovery of order in each domain after prolonged stimulation (as expected from the recovery of the overall membrane order—Fig 3b), but again not of the same magnitude, and resulting in order values for both domains that were distinct from starting conditions.

A likely reason for the observed changes in PM properties during insulin secretion is compositional flux, i.e. membrane remodeling by fusion of the granular membrane, possibly followed by selective re-endocytosis of order-promoting lipids. Indeed, PM cholesterol (relative to other phospholipids) increased by ~20% upon stimulation of the Ins1 cells with glucose and recovered after long-term stimulation (Fig 3d). These data demonstrate that membrane subdomains with a variety of distinct physical properties can be formed in biological membranes, and that the physical phenotypes of these domains can be regulated by compositional variation resulting from cellular activity.

### Disparity in interdomain lipid packing correlates with probe partitioning

Having shown that biomimetic and biologically derived membranes have the potential to form a large number of domains with distinct physical properties (Figs 1 and 2), and that such domains can be generated by cellular activity (Fig 3), we addressed the functional consequences of variable lipid packing by investigating its role on the partitioning and bioactivity of membrane molecules. Membrane domains can regulate cell function by specifically recruiting certain membrane molecules while excluding others. Such a mechanism could concentrate interacting molecules into a “reaction platform” or exclude negative regulators for dynamic regulation of cell signaling [32–34]. Central to this capability is the preferential partitioning of specific membrane molecules between coexisting membrane domains. Although some aspects of membrane domain association (e.g. post-translational lipidation targeting protein to raft domains) have been elucidated [12,35–38], most of the determinants of the phase partitioning of membrane molecules remain unknown.

**Fig 4:**
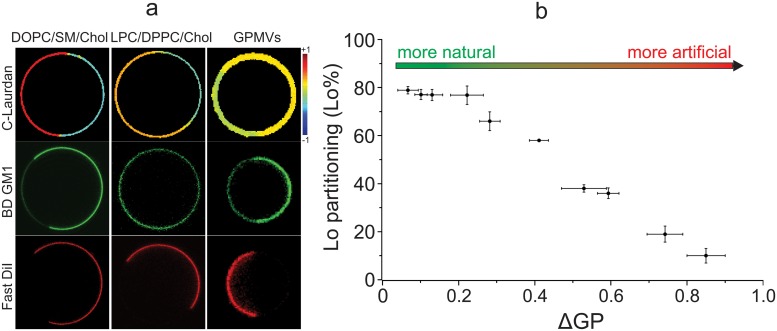
Relative lipid packing between domains correlates with BD-GM1 partitioning. a) GP, BD-GM1 (green) and the disordered marker FAST-DiI (red) imaged in various membrane preparations. BD-GM1 and FAST-DiI are imaged in the same vesicle; GP is shown form a different vesicle representative of the preparation. b) The quantification of BD-GM1 ordered phase partitioning versus ΔGP.

It has been previously observed that partitioning is dependent on both the structure of the partitioning molecule and the membrane in which it is dissolved, i.e. that the same molecule can partition differently in different phase separated mixtures [12,37,39]. We hypothesized that the basis of differential partitioning is the differential lipid packing of membrane domains observed in Fig 2. To test this hypothesis, we assayed the partitioning of a fluorescently labeled analog of the ganglioside GM1 (labeled with Bodipy on the 5^th^ carbon of the sn-2 acyl chain for visualization; BD-GM1) between the coexisting domains of a variety of membrane models. As expected from the inherent affinity of glycosphingolipids for raft domains, BD-GM1 preferentially partitioned to the ordered domain of GPMVs (Fig 4a; column 3). However, the same lipid probe was strongly excluded from the ordered domain of DOPC:SM:Chol GUVs, which present a high order disparity between the coexisting domains (Fig 2). Comparing ΔGP and ordered phase partitioning (quantified as in ref [12]) across all membrane preparations, we observe a clear, inverse relationship, with more biomimetic membranes (i.e. those with smaller order differentials between domains) resulting in more “native” partitioning of BD-GM1 to the ordered domain (Fig 4b).

### Lipid packing disparity regulates membrane receptor-ligand interactions

While BD-GM1 is preferentially targeted to the disordered phase in DOPC:SM:Chol GUVs, there is still a significant proportion that partitions to the ordered phase (%Lo ≈ 10%) [12]. Interestingly, Cholera Toxin B (CTxB), a natural ligand for GM1, recognizes *exclusively* the disordered pool of BD-GM1, showing no binding to the ordered phase (12). We investigated the effect of the spectrum of organizational states accessible to biomimetic membranes on receptor-ligand interaction in the context of GM1 recognition by CTxB.

**Fig 5:**
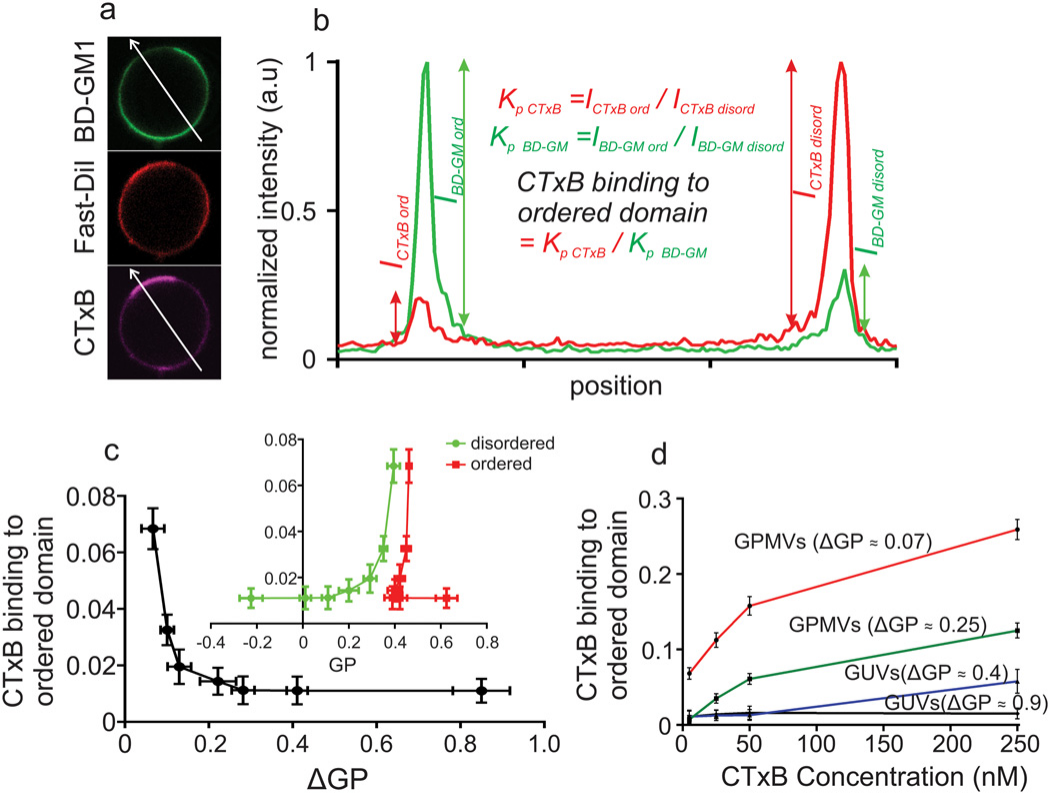
Membrane packing affects receptor-ligand binding. (a) CTxB binds (magenta) almost exclusively to the disordered domain pool of BD-GM1 (green) in phase separated GUVs and GPMVs where disordered phase is marked by FastDiI (red). (b) This behavior is quantified by the “CTxB binding to ordered domain”, the ratio between the phase localization of CTxB (K_p,CTxB_) and its receptor BD-GM1 (K_p,GM1_). (c) At high ΔGP, CTxB essentially does not recognize BD-GM1 present in the ordered domain; as the order difference between phases is reduced, recognition of CTxB in ordered phase increases. CTxB ordered binding is also well-correlated with disordered phase GP but not ordered phase GP. (d) Disordered domain binding of CTxB is preferential, but not exclusive, as ordered phase binding increases with increasing CTxB concentration for lower values of ΔGP.

We assayed two distinct synthetic membrane compositions: DOPC:SM:Chol (2:2:1) representing maximally ordered/disordered Lo/Ld phases yielding the highest inter-domain disparities (ΔGP) in our observations (0.85); and LPC:DPPC:Chol (2:2:1) representing the artificial system with the minimum observed ΔGP (0.43). GUVs doped with 0.1 mol% BD-GM1 were treated with 5 nM CTxB (labeled with Alexa 647) to test ordered phase binding (Fig 5a; 0.1 mol% FAST DiI was included to visualize the disordered phase). Very little CTxB bound to the ordered phase of either synthetic lipid mixture (Fig 5a–5c), despite nearly 40% of the receptor (BD-GM1) partitioning to that phase in the LPC:DPPC:Chol system. The inability to bind ordered phase BD-GM1 extended also to natural membranes, with GPMVs (obtained from CHO cells containing no native GM1 [12]) showing negligible ordered phase CTxB binding (Fig 5c). This result is remarkable because in these GPMVs, BD-GM1 is highly enriched (up to 80%) in the ordered domains (Fig 4b). Only GPMVs designed to minimize the order differences between the domains (ΔGP < 0.1) had any appreciable order phase binding. Fig 5c shows ordered domain binding of CTxB normalized by the relative enrichment/depletion of BD-GM1 in that phase, quantified by a ratio of the partition coefficients (as shown in Fig 5b). If there was no selection of specific BD-GM1 pools by CTxB, ordered domain binding ratio of CTxB would be equal to 1 (partitioning of CTxB = partitioning of BD-GM1). At low CTxB concentration, highly preferential binding of the disordered phase was observed in all membranes (Fig 5c), with appreciable binding of CTxB to the ordered domains only when the differences between the domains were minimized (ΔGP < 0.1). Moreover, we correlated CTxB ordered phase binding preference with the absolute GP of both ordered or disordered phases, rather than the relative difference between the two (ΔGP). While there was no discernible trend between ordered phase GP and CTxB binding (Fig 5c), the disordered phase GP correlated fairly well with ordered phase binding (Fig 5c). However, we believe ΔGP is the more relevant parameter, because overall CTxB binding preference is driven by a binding equilibrium between BD-GM1 in both ordered and disordered domains.

While disordered phase binding was preferred, it was not exclusive, as higher CTxB concentrations eventually lead to an increase in binding of ordered phase BD-GM1, presumably due to saturation of the ‘preferred’ receptor embedded in the disordered domains (Fig 5d). Even under saturating CTxB conditions, there remained a population of BD-GM1 that was unbound by CTxB, as evidenced by <1 CTxB ordered domain binding in all conditions. We note that CTxB binds GM1 in a pentameric fashion; therefore, a linear relationship between receptor concentration and ligand binding is not expected. Nevertheless, the lack of Lo phase binding even when BD-GM1 is highly concentrated in the Lo phase demonstrates that this effect cannot account for our observations. They are also unlikely to be explained by FRET between BD-GM1 and Alexa-CTxB or self-quenching of BD-GM1. In the case of FRET, there is minimal spectral overlap between the two fluorophores (BD-FL emission maximum ~ 510nm; Alexa 647 excitation maximum = 647 nm) and they are physically separated by BD being on the acyl chain of GM1. Self-quenching could occur, but would be more likely in the more BD-GM1 rich ordered phase. Most importantly, the partitioning values for BD-GM1 measured in the presence of CTxB were fully consistent with those without CTxB (shown in Fig 4), confirming that CTxB binding had no effect on real or apparent BD-GM1 partitioning.

We emphasize that BD-GM1 likely does not completely recapitulate the partitioning and binding properties of native, unlabeled GM1 due to the presence of the bulky and somewhat hydrophilic fluorescent probe on the acyl chain. Indeed, based on experiments in GM1 containing cells [12], we expect that native GM1 is strongly Lo partitioning and avidly binds CTxB in that phase. Nevertheless, these data confirm that the bioactivity of membrane-embedded molecules can be regulated by the physicochemical properties of coexisting membrane domains. This effect is clearly demonstrated for CTxB binding BD-GM1 in GPMVs, where the binding activity is modulated by lipid packing (Fig 5c and 5d) despite no difference in receptor partitioning between coexisting domains (first four points in Fig 4b). We hypothesize that the preferential binding of CTxB to BD-GM1 in the Ld phase reflects a distinct conformation of the glycolipid in that domain, as in ref (39). Thus, in addition to concentrating or segregating signaling molecules, membrane domains may direct cell function by regulating the activity of membrane-bound receptors and thereby mediating signal output.

## Conclusion

Cellular membranes are laterally heterogeneous with respect to function and composition, with membrane rafts as the archetypal lipid-driven plasma membrane domains. Here, we observed that both biomimetic and biological membranes phase separate into coexisting domain that access a number of distinct lipid packing states (Figs 1 and 2), and that live cells sample several of these domain states during cellular activity (Fig 3). Microscopic phase separation was observed despite minor differences in local membrane order, quantified by Generalized Polarization (GP). The most dramatic and biologically relevant consequences were the distinct partitioning of functional components between coexisting domains (Fig 4), and the modulation of interaction between membrane components and protein ligands by the lipid packing state (Fig 5).

It is plausible that cells are able to simultaneously access multiple organizational states due to energy-dependent activities such as active lipid turnover, membrane traffic, enzymatic activity, etc. The most obvious examples are distinct membrane compartments (e.g. PM compared to endosomes), which have divergent compositions [40,41], but retain the necessary ingredients for lipid-mediated domain formation (cholesterol, saturated and unsaturated lipids, glycosphingolipids). These distinct compositions potentially support organelle-specific domains of unique compositions and physical properties. More speculatively, local cellular processes may generate conditions that support the coexistence of several discrete domain types within a single contiguous membrane. Access to a tunable variety of organizational states—either in a single membrane or distinct subcompartments—presumably expands membrane functionality by allowing finer control of domain composition, physical properties, and regulation of lipid activity. Finally, domains with variable lipid packing may explain the divergent raft composition/characteristics obtained by different experimental modalities [42], or between detergent-resistant membranes derived with various detergents [6,43].

In summary, our results support a model of biological membranes as a mosaic of raft and non-raft domains with a range of properties and compositions, driven by the preferential interactions between lipids/proteins and regulated by cellular activity. The physical properties of these domains in a given cell or a given membrane remain to be determined, as does the nature of the domain boundaries. The potential diversity of cell membrane domains may shed light on the impressive complexity and flexibility of mammalian membranes recently revealed by quantitative characterization of biological membrane lipidomes [25]. Although the functional impact of this diversity has yet to be elucidated, it is possible that the hundreds of distinct lipid species present in even individual membranes [44] are required to maintain the precise mosaic organization required for a given membrane’s physiology.
